# Polymorphous low-grade neuroepithelial tumor of the young: case report and review focus on the radiological features and genetic alterations

**DOI:** 10.1186/s12883-020-01679-3

**Published:** 2020-04-06

**Authors:** Yingqian Chen, Tian Tian, Xinwen Guo, Fenfen Zhang, Miao Fan, Huawei Jin, Dawei Liu

**Affiliations:** 1 Department of Radiology, the First Affiliated Hospital of Sun Yat-sen University, Guangzhou, Guangdong China; 2 Department of Pathology, the First Affiliated Hospital of Sun Yat-sen University, 58th, The Second Zhongshan Road, Guangzhou, Guangdong China; 3 Psychiatric Department, Guangdong 999 Brain Hospital, Guangzhou, Guangdong China; 4 Department of Neurosurgery, the First Affiliated Hospital of Sun Yat-sen University, Guangzhou, Guangdong China

**Keywords:** Polymorphous low-grade neuroepithelial tumor of the young, MRI, CT, Pathology, Genetic alterations

## Abstract

**Background:**

A new type of epileptogenic tumor, the polymorphous low-grade neuroepithelial tumor of the young (PLNTY) was firstly reported by Jason T. Huse et al. at 2016. After that, only 1 case of PLNTY was reported by article. The radiological characteristics of PLNTY have not been concluded. The objective of our study was to report 3 cases of PLNTYs in details and to analyze the image characteristics and genetic alterations of PLNTYs by reviewing our cases and articles.

**Case presentation:**

There were 3 cases diagnosed as PLNTY by pathology in our hospital during the last 10 years, with the average age of 15. They were all suffered from different degrees of epilepsy. All of them underwent magnetic resonance (MR) imaging and 2 of them underwent computer tomography (CT) imaging. The PLNTYs are all appearing as a solid or solid-cystic cortical mass with little mass effect and unclear boundary with normal brain tissue. They are all shown as hyperintensity in T2WI and iso−/hypointensity in T1WI with slight or no enhancement after contract enhanced in MR imaging. The “salt and pepper sign” in T2WI and grit calcification in CT images might be specific characteristics of PLNTY. All of them recovered after excision of the tumors. The gene tests revealed fibroblast growth factor receptors 3 (FGFR3)-TACC3 fusion and FGFR3 amplification in one case, and the B-Raf proto-oncogene (BRAF) V600E mutation in another case.

**Conclusion:**

In the image, the partial ill-marginated cortical mass with “salt and pepper sign” in T2WI or grit calcification in CT imaging might be the typical imaging characteristics of PLNTY. We also prove that the BRAF V600E mutation as well as the FGFR2 and FGFR3 have a close relationship with PLNTY.

## Background

Low grade neuroepithelial neoplasm commonly presents with clinical seizure onset on early life, which is a group of brain heterogeneous tumours with a broad spectrum and variable histomorphological and genetic alteration features. Of them, the common entities include dysembryoplastic neuroepithelial tumors (DNET), gangliogliomas, oligodendroglioma, pleomorphic xanthoastrocytoma, infantile low-grade glioma and so on [1, 2]. In 2016, Jason T. Huse et al. reported a new type of epileptogenic tumor and named the entity as the polymorphous low-grade neuroepithelial tumor of the young (PLNTY) [3], a distinct epileptogenic neoplasm, characterized morphologically and molecularly by the presence of oligodendroglioma-like cellular component and genetic abnormalities of either B-Raf proto-oncogene (BRAF) or fibroblast growth factor receptors 2 and 3 (FGFR2, FGFR3). However, the radiological characteristics of PLNTY have not been reported yet. Here we report three classic cases of PLNTYs and review articles in the literatures, and aim to conclude the radiological characteristics and genetic alterations of PLNTY.

## Case presentation

### Case 1

A 14-year-old girl with seizure for 1 year was referred to the hospital with the stable symptoms of confusion, twitching limbs, deviated mouth, cyanosis of lips, urinary and fecal incontinence when the epilepsy happened. The EEG found out the epileptoid discharge in the left temporal lobe. MRI showed an irregular lesion with partial blurry boundary located in the left temporal lobe. The lesion appeared as relative hypointensity in T1WI and hyperintensity in T2WI with the “salt and pepper sign”, with slightly enhancement. Then she received surgery. At gross specimen inspection, the tumor was seen as a soft mass with greyish white section. At microscopy, the tumor exhibited oligodendroglioma-like cellular component with moderate polymorphic nuclei and multiple grit calcification. Immunolabeling for Olig2, Vimentin, CD34, S-100, Syn were positive; and GFAP, NF, IDH1, P53, NeuN, EMA, B-raf were negative; Ki67 < 1%. Total RNA extracted from Formalin-fixed and paraffin-embedded (FFPE) material was analyzed for fusion transcripts using anchored multiplex polymerase chain reaction (PCR)-based methodology (ArcherDx) and revealed FGFR3-TACC3 fusion in this case [4]. Moreover comprehensive DNA sequencing analyses identified the FGFR3 amplification in the tumor. Focal cortical dyspliasia (FCD) Ib was also be found in the periferal area of the mass. The epilepsy sympton didn’t happen again under the control of anti-epilepsy medicine in the 3 months’ follow-up.

### Case 2

A 15-year-old boy was admitted to hospital with the chief complain of repeated tetany of the left upper limb with loss of consciousness for half a year. The head CT was done and showed a mass with grit calcification located in the right temporal lobe. Then he was transferred to our hospital and receive the brain MR, which showed a mass located in the right temporal lobe with clear boundary, which appears isointensity in T1WI, hyperintensity in T2WI and T2-FLAIR sequence and slightly patchy enhancement. The “salt and pepper sign” could also be seen in T2WI. After the surgery, pathology proved the mass to be combined with uniform oligodendroglioma-like component with discrete calcospherules. And the immunolabeling for CD34, Vimentin, and Olig-2 were positive, but negative for IDH-1, EMA, SOX10, NeuN, EMA, P53and MGMT. The B-raf V600E mutaion was identified in this case by molecular test with mutation-specific immunohistochemical staining. This patient was totally free from the epilepsy symptoms under the control of anti-epilepsy medicine in the 3 months’ follow-up.

### Case 3

A 16-year-old boy suffered from paroxysmal convulsion of angulus oris for 2 years and progressed to the whole body for half a year. A non-enhanced CT imaging showed a solitary-cystic mass with hyperattenuation calcification located in the right frontal lobe. Then he underwent conventional MRI scanning including diffusion weighted imaging (DWI), diffusion tensor imaging (DTI) and H1-magnetic resonance spectrum (H1-MRS). MR imaging revealed a solitary-cystic mass with uniform T1WI signal, slight increased T2WI signal with “salt and pepper sign” and slightly enhancement of the solitary part. DWI proved the mass did not have restricted diffusion. In DTI, the white matter fiber tracts appeared as being slightly compressed due to the small range of peripheral edema but still being integrated. H1-MRS demonstrated a slightly declined NAA (N-Acetyl Aspartate) peak and an elevated Cho (Choline) peak, which revealed slight decrease of nerve cell and increase of the gliocytes. He received surgery and the mass was removed completely. At gross specimen inspection, the mass appeared as a unencapsulated gray-white mass with unclear boundary of peripheral brain tissue. Under microscopy, the tumor showed infiltrative growth pattern with predominant oligodendroglioma-like component with moderate cellular pleomorphism, and absence of both tumor necrosis and mitosis. The immunostaining test showed that the tumor was positive for Vimentin, CD34, and Olig-2, but negative for IDH-1, EMA, SOX10, NeuN, EMA, P53and MGMT. It was the pity that this case didn’t get the gene tests. Till the follow-up in 36 months after the surgery, the patient still was free from the epilepsy without medicine.

All of the cases have completed central pathological review and met the PLNTY criteria of the following: 1) infiltrative and nodular growth pattern; 2) invariable presence of oligodendroglioma-like cellular component with more or less polymorphic cellular elements including pleomorphic and spindle cells; 3) intense immunolabeling for cluster of differentiation 34 (CD34) [3].

**Fig. 1:**
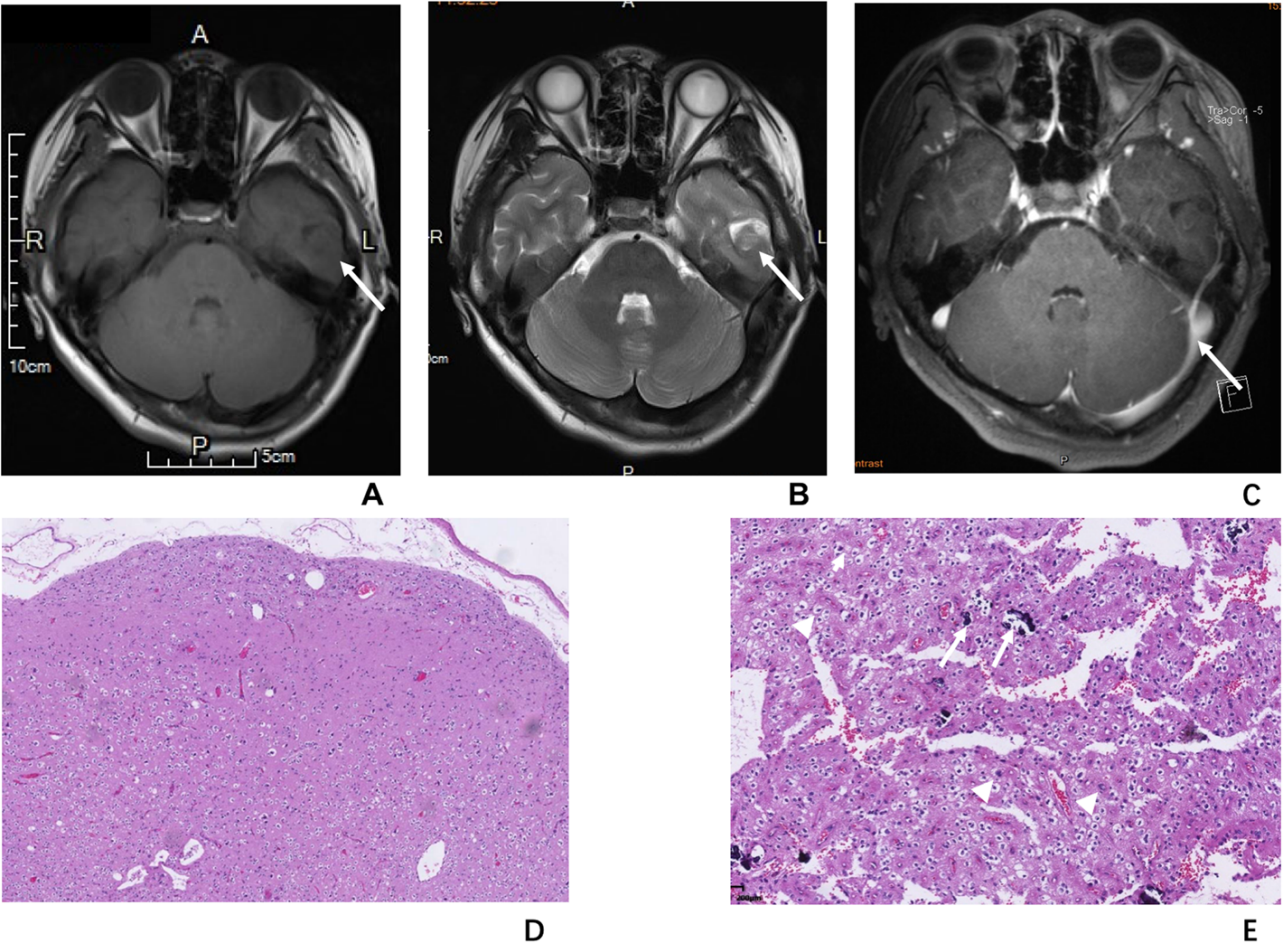
The MR images and pathological images of a 14-year-old girl with PLNTY in the left temporal lobe. The MR images and pathological images of a 14-year-old girl with PLNTY in the left temporal lobe. **a**, The tumor (arrow) appeared as clear-marginated cortical mass with slightly hypointensity in T1WI. **b**, The tumor (arrow) showed hyperintensity with “salt and pepper sign” in T2WI. **c**, The tumor (arrow) showed no enhancement after contrast agent injection. **d** (H-E staining,40×), Under microscopy, the tumor, located in the cerebral cortex and superficial white matter, showed an infiltrative growth pattern with adjacent tissue involvement and subpial extension. **e** (H-E staining, 200×), Oligodendrocyte-like-cells (short arrow), astrocytes, and spindle cells with moderate nuclear pleomorphism (arrow heads) and scattered calcifications (arrows) can be seen inside the tumor

**Fig. 2:**
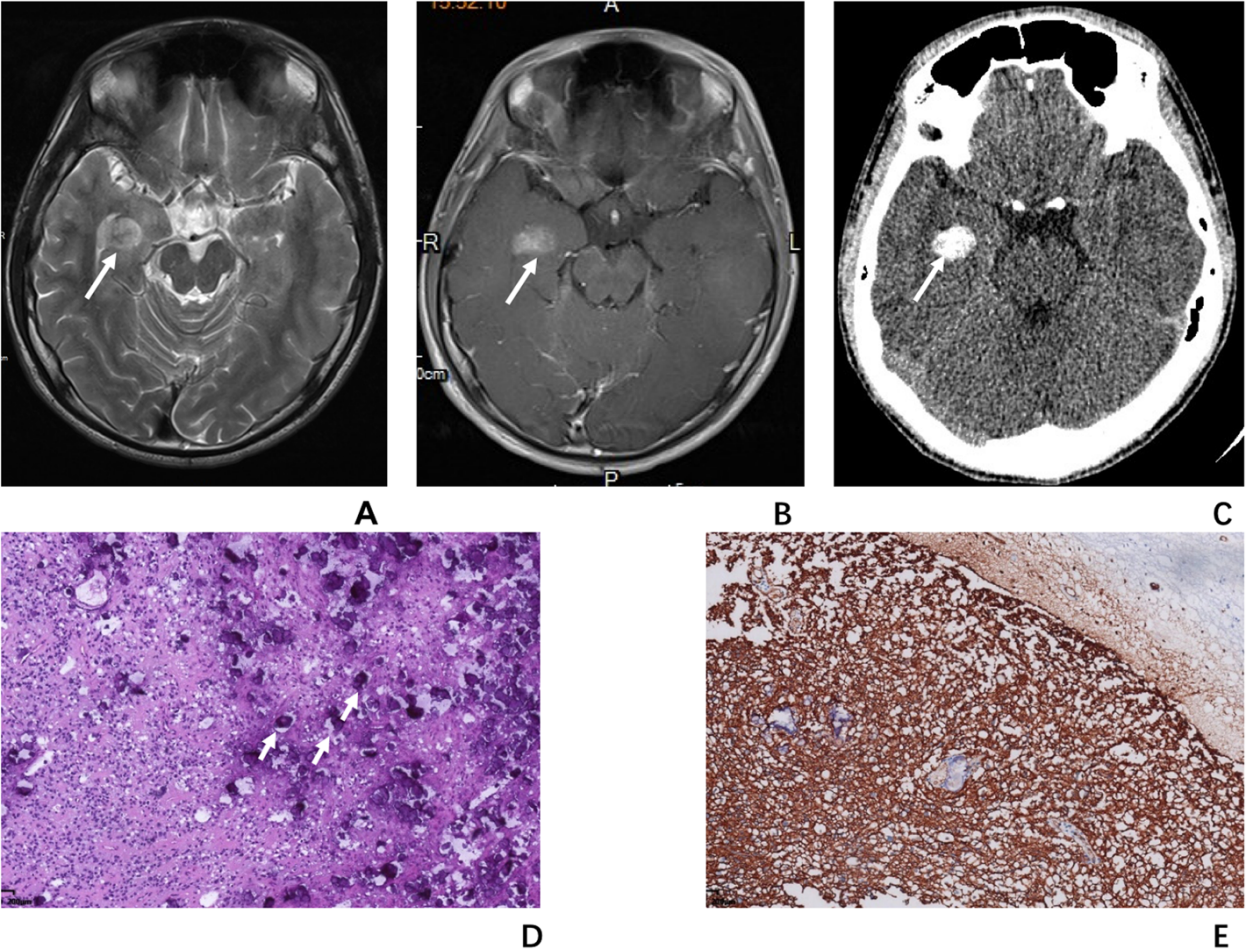
The MR, CT images and pathological images of a 15-year-old boy with PLNTY in the right temporal lobe. **a**, The tumor (arrow) appeared as a cortical-originated hyperintensity mass with “sign” in T2WI. **b**, The tumor (arrow) showed patchy enhancement in T1WI after gadolinium contrast administration. **c**, Thick calcification (arrows) can be seen in CT image. **d** (H-E staining,200×), Under microscopy, the tumor showed discrete calcospherules to confluent calcific masses (short arrows). **e** (CD34 staining, 100×), The immunolabeling for CD34 was intense and widespread

The CT, MR and pathology images are shown in Figs. 1 and 2.

## Discussion and conclusion

A heterogenous group of brain tumors is associated with early epilepsy in children and young adults. The vast majority of these tumours are low grade (WHO grade I or WHO gradeII) brain tumor, and exhibit large spectrum of morphological variants. The common recognized entities are called long-term epilepsy associated brain tumours (LEAT), which consist of pilocytic astrocytoma, ganglioglioma, pleomorphic xanthoastrocytoma (PXA), DNET, angiocentric glioma, pediatric oligodendroglioma and so on [5] . Most of them do not share the molecular features typically observed in adult diffuse gliomas, such as IDH1 mutations or 1p/19q co-deletions. But they commonly involve some genes associated with embryonic development. And they are usually identified to have the expression of oncofoetal marker protein CD34 by immunohistochemistry. Genetic alterations involving MAPK pathway (especially BRAF V600E mutation) and mTOR signaling pathway may be key genetic features in this group of tumours [6]. In 2016, Jason T. Huse et al. firstly reported a new entity named PLNTY, and defined distinct pathological features including the presence of oligodendroglioma-like cellular components, an infiltrative growth pattern, and intense CD34 immunopositivity [3]. Clinically, according to the description in the article, PLNTYs often occur in younger patients in association with epileptogenic activity, tend to exhibit a benign (WHO grade I) clinical course, and appear to be well controlled by gross total resection. Both clinic data and molecular profiling indicate that PLNTY is a new entity belonging to neoplasm associated with early epilepsy onset. Few PLNTY cases of following reported articles yield the insufficient knowledge of radiology to apply to the diagnostic setting [7]. Furthermore, radiologists can easily misdiagnose or underdiagnose this new entity. And sometimes it is very difficult to distinguish form other entities with similar features. In the present study, we evaluate three cases of PLNTYs and provide a detailed description of radiological characteristics and genetic alteration related to MAP signaling pathway activation including BRAF V600E mutation, FGFR arrangement.

The clinical features and locations of the three cases are assembled and shown in Table 1. The main clinical features are similar in each case and concordant with previous reports, including presence of the earlier seizures, slow progressing, and affecting children and young adults (from14 to 16 years old). In the 14 diagnosed cases (including 3 cases of ours), the average age is 18 (range from 4 to 32 years). The ratio of male to female is 1:1. Most of the patient received gross total tumor removal (11/12) and recovered from seizure (10/11). In addition, with reviewing the research on cases reported in other series, we tried to conclude the radiological characteristics and genetic alterations of PLNTY and got a deeper understanding the new kind of epileptogenic tumor. Pathologically, all our three cases are composed of invariably characteristic oligodendroglioma-like cellular components, and the presence of intense CD34 immunopositivity. Huse et al. also provided the CT or MR images of 4 cases. Concluded by our cases and cases reported by Huse, PLNTYs are all originated form the cortex layer, appearing as a solid or solid-cystic mass with little mass effect and unclear boundary with normal brain tissue. Most of them are located in the temporal lobes (8/11 from the reports and 2/3 in our cases), followed by the occipital lobe and frontal lobe. They are all shown as hyperintensity in T2WI and iso- or hypointensity in T1WI with slight or no enhancement after contract enhanced. All the 6 cases with CT image showed the lesion-associated grit calcification. The “salt and pepper sign”, which represents the granulate mixed signals in T2WI and might be a specific characteristic of PLNTY (6/6), is potentially due to the grit calcification. One of our cases still showed a slight decrease of NAA and increase of Cho in H^1^-MRS, no restricted diffusion in DWI, and compression rather than disruption of the fiber tracts in DTI, which all suggest the PLNTY to be a low grade, less aggressive neoplasm.

**Table 1:** The basic clinical data, imaging characteristics and genetic alterations of PLNTY

case	Age	Gender	Presentation	Location	Treatment	Outcome	“salt and pepper sign” in T2WI	Calcification	BRAF mutation	Fusion transcripts
**1**	14	F	Focal epilepsy	L temporal	GTR	NED	Pos	Pos	Neg	FGFR3-TACC3
**2**	15	M	Focal epilepsy	R temporal	GTR	NED	Pos	Pos	BRAF V600E	
**3**	16	M	Focal epilepsy	R frontal	GTR	NED	Pos	Pos	N/A	
**4 [7]**	31	M	Focal epilepsy	Temporal	N/A	N/A	N/A	N/A	BRAF V600E	
**5 [3]**	16	M	Epilepsy	R temporal	GTR	NED	Pos	Pos in 9/10 cases	BRAF V600E	
**6 [3]**	18	F	Epilepsy	R temporal	STR	Post-op psychosis	Pos		BRAF V600E	
**7 [3]**	23	F	Epilepsy	R temporal	N/A	N/A	N/A		BRAF V600E	
**8 [3]**	17	F	Epilepsy	R temporal	GTR	NED	N/A		Neg	FGFR3-TACC3
**9 [3]**	4	M	Epilepsy	L temporal	GTR	NED	N/A		Neg	FGFR2-CTNNA3
**10 [3]**	9	M	Epilepsy	R frontal	GTR	New seizures after 36 months	Pos		Neg	FGFR2-KIAA1598
**11 [3]**	10	M	Dizziness	R occipital	GTR	NED	N/A		Neg	FGFR2-KIAA1598
**12 [3]**	23	F	Epilepsy	R temporal	GTR	NED	N/A		Neg	
**13 [3]**	32	F	Epilepsy	R temporal	GTR	NED	N/A		N/A	
**14 [3]**	24	F	Visual disturbances	R temporal	GTR	NED	N/A		N/A	

*NED* No Evidence of Disease, *GTR* Gross Total Tumor Removal, *STR* Simple Tumor Removal

The pathologic features of PLNTY also support the imaging characteristics. Two (case 1 and 3) of them prominently show an infiltrative growth pattern with adjacent tissue involvement and subpial extension, and partly exhibit sharp borderline. And case 2 has a completely well-defined borderline. The infiltrated growth patterns may explain the unclear boundary with normal brain tissue in radiology. Grit calcification can be seen in 12 of 13 cases with pathology reports, which support the relevant sign in CT and MR imaging.

As reported in the seminal article by Jason T. Huse et al., most of the PLNTYs had either BRAF alternation or FGFR fusion. BRAF is a crucial regulator of the MAPK pathway. And FGFR is an upstream regulator. The mutations these genes will all involve the MAPK oncogenic pathway, just like the other pediatric low-grade gliomas [8]. In 9 cases of relevant molecular tests, 3 cases were found to have BRAF V600E mutation, and 4 other cases were found to have FGFR2/FGFR3 arrangement. Also, gene alteration analysis was performed in our three cases by using RNA sequencing and PCR. The authors reported two cases of PLNTYs harboring BRAF V600E mutation, and one case identified with FGFR3-TACC3 fusion highlighted as a previously commonly described cases. These tumors displayed a unique nodular pattern composed of oligodendroglioma-like cellular components arranged in pseudorosette like pattern. The tumor cells have constant expression of S100 protein, Olig2, Vimentin, CD34, resembling other neoplasms associated with early epilepsy onset. Necrosis and a high mitotic activity were no observed in all cases. For non-specific for PLNTYS, the abnormal MAPK pathway activation is generally founded in many other LEATs including DNET, pilocytic astrocytoma, FCD, ganglioglioma and PXA. The similar molecular origin may partially explain the similar imaging morphology of these tumors. The basic clinical data, imaging characteristics and genetic alterations are listed in Table 1.

**Fig. 3:**
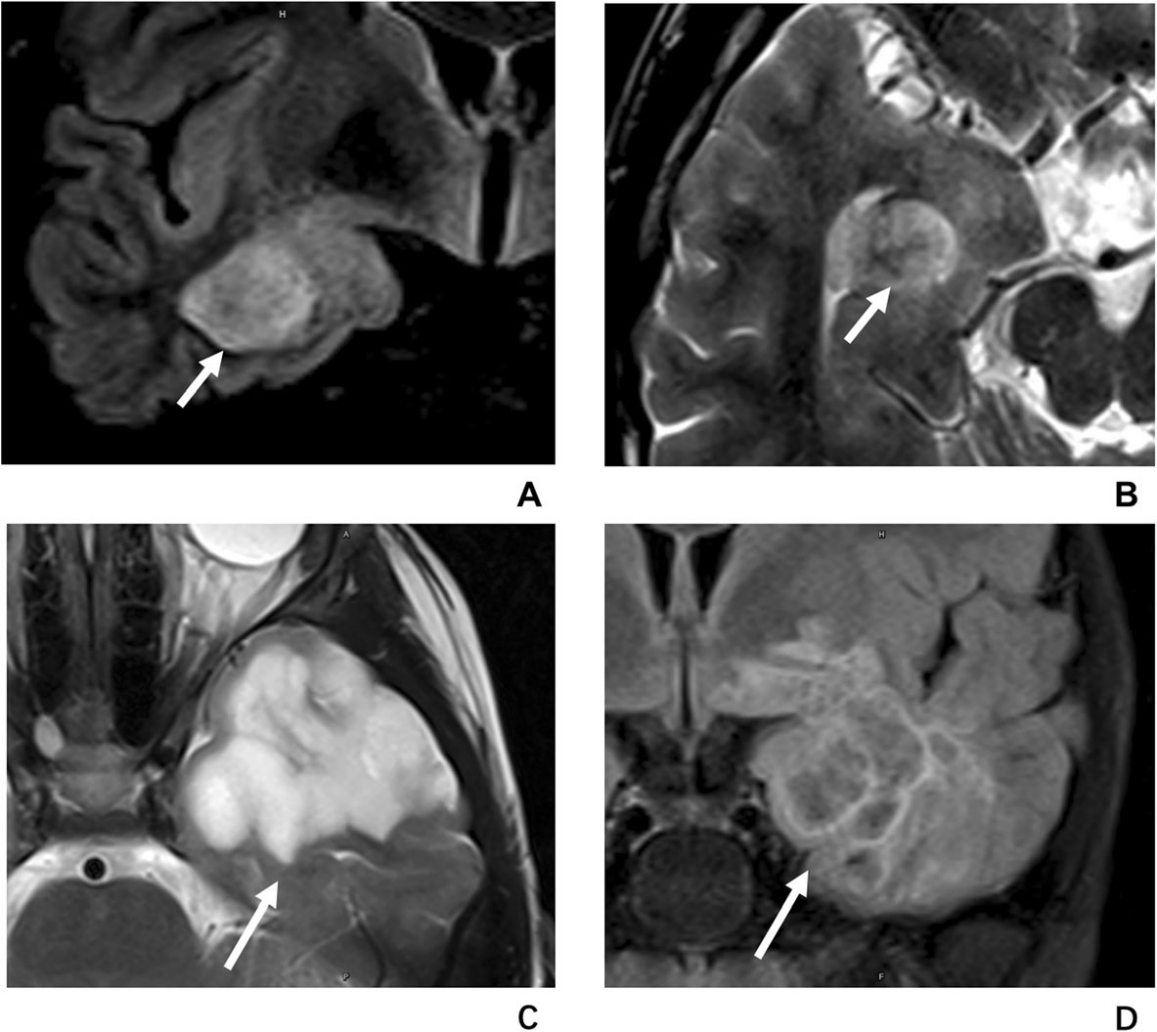
The comparison between “salt and pepper sign” in PLNTY and “soap bubble sign” in DNET. The T2WI (**a**) and T2-FLAIR (**b**) images of a case of PLNTY show the “salt and pepper sign” (short arrow) caused by grit calcification. The “soap bubble sign” (arrow) which represents the multiple cystic lesion can be seen in the T2WI (**c**) and T2-FLARI (**d**) images of a 15-year-old boy with DNET

By reviewing all LEATs in our cohort, we found all PLNTYs were misdiagnosed as DNET, in that both having a similar appearance in both radiology and pathology. Indeed, PLNTY and DNET has many characteristics in common. As the epileptogenic tumors, the DNET and PLNTY are all characterized by a predominantly cortical location and by drug-resistant partial seizures as the clinical manifestation [9]. They both tend to be found in children or young adults, mostly because of their embryonic origin. PLNTY and DNET also have many common features in imaging. They were all originated form the cortex layer and have similar intensity change in T1WI, T2WI and contract-enhanced T1WI. While PLNTY and DNET have some different imaging characteristics. Form our cases and the other cases reported, we found that the size of PLNTYs (average diameter of 20 mm in our cases and 0.7 to 3 cm from the reports) are smaller than the DNET (most of them are lager than 3 cm) [10]. What’s more, DNET tends to have a clear demarcation and multinodular appearance like “soap-bubble” in MR imaging [9]. The comparison between the “salt and pepper sign” in PLNTY and “soap bubble sign” in DNET is shown in Fig. 3. Fine calcifications are generally observed in DNET but does not appear as “salt and pepper sign”. Moreover, DNET was reported to have the potential possibility of malignant transformation, which has not be found in PLNTY [9].

The other differential diagnoses should include other cortical originated lesions like FCD, gangliogliomas and oligodendroglioma. FCD is another major cause of drug-resistant epilepsy. Its imaging features are varying. In general, the extent of the lesion is smaller than the other epileptogenic tumors. Focal cortical thickening, blurry gray white matter interface, focally increased signal on T2WI and the “transmantle” sign are the MR characteristics of FCD [11, 12]. Interestingly, FCD Ib was observed in the transition region in one of our cases, which might imply the homogeneous genetic origination of PLNTY and FCD.

Gangliogliomas is another common tumor which can cause chronic temporal lobe epilepsy in adolescent. Typical gangliogliomas showed as cystic-solitary tumor with ring-enhancement or solid-enhancement and arc calcification. Specially, it always appears as isointense to the gray matter in T1WI [13]. The prominent cystic component, different morphology of calcification and specific T1 signal intensity can help for differentiation diagnosis with PLNTY.

Oligodendroglioma is a kind of WHO grade II epilepsy-associate tumor. But it is predominantly occurring in adults than in children or adolescents. It has a cortical-subcortical location and mostly located in the frontal lobe. Gyriform calcification is a characteristic of oligodendroglioma. Though it can also appear as iso- or hypointensity in T1WI and heterogeneous hyperintensity in T2WI as the other cortical-based tumors, minimal to moderate enhancement and increased perfusion can distinguish it from other low-grade gliomas [14, 15]. Testing IDH mutation and 1p/19q codeletion can conformed the diagnosis.

In summary, we have reported 3 cases of a distinctive, low grade neuroepithelial tumor associated with MAPK signal pathway, and propose the radiological features. Our cases and current reported cases suggest that the partial ill-marginated cortical mass with hyperintensity with “salt and pepper sign” in T2WI or grit calcification in CT imaging might be the typical imaging characteristics of PLNTY. And classical genetic alterations including the BRAF 600E mutation as well as the FGFR2/FGFR3, have been found close relationship with PLNTY, which are commonly seen in LEATs. The clinical behavior of the three current described cases of PLNTYs was non-aggressive with no evidence of recurrence or metastasis (follow-up ranged from 6 to 10 months). This tumor should be considered a low-grade malignancy (WHO gradeI). We hope that this report prompts others to recognize similar tumors helping to create a more complete understanding on their radiology and molecular genetics.

## Data Availability

We carefully documented all the patients’ data reported in the article. We will share the de-identified data at the request of other qualified investigators for purposes of scientific research or teaching. To request the data, please contact the corresponding authors.

## Abbreviations

PLNTY: Polymorphous low-grade neuroepithelial tumor of the young
CT: Computer tomography
MR: Magnetic resonance
BRAF: B-Raf proto-oncogene
FGFR: Fibroblast growth factor receptors
DNET: Dysembryoplastic neuroepithelial tumor
DWI: Diffusion weighted imaging
DTI: Diffusion tensor imaging
H1-MRS: H1-magnetic resonance spectrum
FCD: Focal cortical dyspliasia
LEAT: Long-term epilepsy associated brain tumours
PXA: Pleomorphic xanthoastrocytoma
PCR: Polymerase chain reaction
